# The Relationship Between Daily Rapid-Eye Movement Sleep and Cognitive Functioning in Older Adults

**DOI:** 10.1093/geroni/igaa057.518

**Published:** 2020-12-16

**Authors:** Tiana Broen, Tomiko Yoneda, Jonathan Rush, Jamie Knight, Nathan Lewis, Rebecca Vendittelli, Scott Hofer, Andrea Piccinin

**Affiliations:** 1 University of Victoria, Victoria, British Columbia, Canada; 2 University of Victoria, University of Victoria, British Columbia, Canada; 3 Psychology, Victoria, British Columbia, Canada

## Abstract

Previous cross-sectional research suggests that age-related decreases in Rapid-Eye Movement (REM) sleep may contribute to poorer cognitive functioning (CF); however, few studies have examined the relationship at the intraindividual level by measuring habitual sleep over multiple days. Applying a 14-day daily diary design, the current study examines the dynamic relationship between REM sleep and CF in 69 healthy older adults (M age=70.8 years, SD=3.37; 73.9% female; 66.6% completed at least an undergraduate degree). A Fitbit device provided actigraphy indices of REM sleep (minutes and percentage of total sleep time), while CF was measured four times daily on a smartphone via ambulatory cognitive tests that captured processing speed and working memory. This research addressed the following questions: At the within-person level, are fluctuations in quantity of REM sleep associated with fluctuations in next day cognitive measures across days? Do individuals who spend more time in REM sleep on average, perform better on cognitive tests than adults who spend less time in REM sleep? A series of multilevel models were fit to examine the extent to which each index of sleep accounted for daily fluctuations in performance on next day cognitive tests. Results indicated that during nights when individuals had more REM sleep minutes than was typical, they performed better on the working memory task the next morning (estimate = -.003, SE = .002, p = .02). These results highlight the impact of REM sleep on CF, and further research may allow for targeted interventions for earlier treatment of sleep-related cognitive impairment.

